# Dissociable effects of methylphenidate, atomoxetine and placebo on regional cerebral blood flow in healthy volunteers at rest: A multi-class pattern recognition approach

**DOI:** 10.1016/j.neuroimage.2012.01.058

**Published:** 2012-04-02

**Authors:** Andre F. Marquand, Owen G. O'Daly, Sara De Simoni, David C. Alsop, R. Paul Maguire, Steven C.R. Williams, Fernando O. Zelaya, Mitul A. Mehta

**Affiliations:** a Department of Neuroimaging, Centre for Neuroimaging Sciences, Institute of Psychiatry, King's College London, UK; b Beth Israel Deaconess Medical Center and Harvard Medical School, Boston, Massachusetts, USA; c Pfizer Global Research and Development, Eastern Point Road, Groton, Connecticut, USA

**Keywords:** Methylphenidate, Atomoxetine, Arterial spin labeling, Multi-class pattern recognition

## Abstract

The stimulant drug methylphenidate (MPH) and the non-stimulant drug atomoxetine (ATX) are both widely used for the treatment of attention deficit/hyperactivity disorder (ADHD), but their differential effects on human brain function are poorly understood. PET and blood oxygen level dependent (BOLD) fMRI have been used to study the effects of MPH and BOLD fMRI is beginning to be used to delineate the effects of MPH and ATX in the context of cognitive tasks. The BOLD signal is a proxy for neuronal activity and is dependent on three physiological parameters: regional cerebral blood flow (rCBF), cerebral metabolic rate of oxygen and cerebral blood volume. To identify areas sensitive to MPH and ATX and assist interpretation of BOLD studies in healthy volunteers and ADHD patients, it is therefore of interest to characterize the effects of these drugs on rCBF. In this study, we used arterial spin labeling (ASL) MRI to measure rCBF non-invasively in healthy volunteers after administration of MPH, ATX or placebo. We employed multi-class pattern recognition (PR) to discriminate the neuronal effects of the drugs, which accurately discriminated all drug conditions from one another and provided activity patterns that precisely localized discriminating brain regions. We showed common and differential effects in cortical and subcortical brain regions. The clearest differential effects were observed in four regions: (i) in the caudate body where MPH but not ATX increased rCBF, (ii) in the midbrain/substantia nigra and (iii) thalamus where MPH increased and ATX decreased rCBF plus (iv) a large region of cerebellar cortex where ATX increased rCBF relative to MPH. Our results demonstrate that combining ASL and PR yields a sensitive method for detecting the effects of these drugs and provides insights into the regional distribution of brain networks potentially modulated by these compounds.

## Highlights

►Methylphenidate and atomoxetine are both commonly prescribed for treatment of ADHD. ►We measured their effects on rCBF in healthy volunteers with arterial spin labeling. ►We employed pattern recognition to discriminate the neuronal effects of the drugs. ►Clear differential effects were found in caudate, cerebellum, midbrain and thalamus. ►Our results are useful to identify brain networks potentially modulated by the drugs.

## Introduction

Pharmacological agents that increase the extracellular concentration of the catecholamines noradrenaline (NA) and dopamine (DA) are commonly prescribed to relieve the symptoms of attention-deficit hyperactivity disorder (ADHD). Methylphenidate (MPH) is a stimulant drug that is the treatment of choice in most cases although the non-stimulant drug atomoxetine (ATX) is also increasingly being used for ADHD treatment. MPH has a greater clinical efficacy than ATX (Faraone et al., 2005; Kemner et al., 2005; Michelson et al., 2001; Newcorn et al., 2008; Spencer et al., 1998; Starr and Kemner, 2005), but ATX offers several advantages over MPH. Most importantly, ATX provides an alternative treatment for patients who do not respond to stimulants, it has a reduced abuse liability and a reduced risk of motor side effects (Biederman et al., 2004; Newcorn et al., 2008). Both drugs exert their primary effects by blocking catecholamine reuptake but they differ in that MPH inhibits both DA and NA transporters (DAT and NAT respectively; Seeman and Madras, 1998; Han and Gu, 2006) whereas ATX is a selective inhibitor of NAT (Bolden-Watson and Richelson, 1993; Wong et al., 1982).

To date, the effects of MPH, but not ATX, have been examined using PET markers of glucose utilization and regional cerebral blood flow (rCBF). These studies have demonstrated consistent increases in relative cerebellar activity (Mehta et al., 2000; Udo de Haes et al., 2007; Volkow et al., 1997) and differential effects on rCBF in the temporal poles (Mehta et al., 2000; Udo de Haes et al., 2007). Udo de Haes et al. (2007) also showed increased rCBF in the anterior cingulate cortex and supplementary motor area and decreases in the superior temporal gyrus, middle frontal gyrus and inferior parietal cortex while Mehta et al. (2000) showed decreased rCBF in middle temporal gyrus, occipital gyrus and the frontal pole. These studies demonstrate that MPH influences blood flow across widespread brain regions which may be due to localized effects of MPH at catecholamine transporter sites or consequent effects on connected brain regions. The relatively small sample sizes of these studies or variations in administered dose of MPH may have contributed to the differences in reported findings. The differences may also reflect a limitation of the univariate analysis approach combined with a fixed significance threshold, which might control the false positive rate, but is not sensitive to similarities in spatially distributed patterns of activity. Therefore, the use of a fixed threshold may have contributed to different features of similar underlying patterns being reported in different studies. In addition to directly characterizing the consistent spatially distributed effects of MPH and ATX on rCBF, the use of analysis methods sensitive to spatially distributed patterns may also be beneficial for discriminating the neuronal effects of MPH, ATX and placebo (PLC), providing a drug condition prediction for each subject and session. In this study, we employed a multi-class pattern recognition (PR) approach for these purposes that enabled simultaneous discrimination of all three drug conditions from one another and provided a parsimonious representation of the differential activity patterns for MPH and ATX (Krishnapuram et al., 2005; Ryali et al., 2010).

In addition to the PET studies noted above, functional neuroimaging has been used to study the effects of MPH and ATX on task networks associated with response inhibition (Chamberlain et al., 2009; Vaidya et al., 1998), error monitoring (Graf et al., 2011; Rubia et al., 2011), reversal learning (Dodds et al., 2008) and working memory (Mehta et al., 2000; Schweitzer et al., 2004). In a previous report, we directly compared the effects of MPH and ATX in the same participants included in the present manuscript while they performed a rewarded working memory task. We reported: (i) that both MPH and ATX attenuate BOLD activity in working memory networks and enhance task-related deactivations during rewarded working memory trials and (ii) that MPH and ATX have opposing effects on activated and deactivated networks during the delay component of rewarded trials (Marquand et al., 2011).

While functional imaging with blood oxygen level-dependent (BOLD) fMRI is appropriate to study relative signal changes between task conditions, it cannot provide specific information about the physiological mechanisms that drive the BOLD response. This is important because changes in local deoxyhaemoglobin concentration (on which the BOLD signal depends directly) are determined by changes in rCBF, regional cerebral metabolic rate of oxygen (rCMRO_2_) and cerebral blood volume (rCBV). Thus, the magnitude of the BOLD response in functional imaging studies depends not only on the baseline changes in rCBF but also other parameters (Buxton, 2010; Buxton et al., 2004). In contrast, arterial spin labeling (ASL; Detre et al., 1992; Williams et al., 1992) is an emerging imaging technique that can measure rCBF quantitatively and non-invasively. In this study, we aimed to characterize the effects of ATX and MPH on rCBF using ASL and PR. These results will be useful to assist interpretation of BOLD findings since they define the pattern of regional rCBF changes produced by MPH and ATX, and to provide insights into the regional distribution of the brain networks potentially modulated by these compounds.

Based on earlier neuroimaging studies we hypothesized that we would be able to accurately discriminate MPH from PLC based on rCBF changes in a network of regions including the cerebellum and temporal poles. Additionally, a rodent microdialysis study has shown differential effects of MPH and ATX on striatal DA levels (Bymaster et al., 2002), thus we also expected the striatum to be an important region for discriminating the two compounds. No studies to date have examined the effects of ATX on brain metabolism or blood flow but the high density of NAT in the locus coeruleus (LC), thalamus, hypothalamus, cerebellum, paracentral lobule and supplementary motor area (Hannestad et al., 2010; Schou et al., 2005; Tejani-Butt, 1992) provides a network of regions that we hypothesized would contribute to the accurate discrimination of ATX from PLC.

## Methods

### Participant recruitment and study design

Fifteen healthy, right-handed male participants (aged 20–39) were recruited by local advertisement and each scanned on three occasions. Exclusion criteria have been described previously (Marquand et al., 2011), but in brief they included any current illnesses, smoking > 5cigarettes per day, consuming > 5cups of coffee per day and any history of psychiatric, neurological problems or substance abuse in addition to conventional MRI exclusion criteria. Participants provided written informed consent and the study was approved by the South London Research Ethics Committee. Participants were asked to refrain from consuming alcohol or caffeine containing products 24 h prior to dosing and on each scanning day participants were screened for drugs of abuse and alcohol. Each participant then received an oral dose of MPH (30 mg), ATX (60 mg), or a PLC according to a randomized, double-blind Latin square design. Doses of MPH and ATX were chosen to approximately match doses commonly used in clinical practice, and doses reported in the literature (e.g. Gilbert et al., 2006). Based on existing human catecholamine transporter occupancy studies, we estimated that 30 mg of oral MPH resulted in approximately 65% DAT occupancy in the striatum and approximately 50% NAT occupancy in the thalamus (Hannestad et al., 2010; Volkow et al., 1998). We did not estimate transporter occupancy for ATX as we are not aware of any studies investigating the relationship between ATX dose and transporter occupancy in humans.

### MRI data acquisition and preprocessing

Scanning was performed on a General Electric Signa HDx 3T scanner and was timed to coincide with the peak plasma concentration for MPH and ATX (Sauer et al., 2005; Wargin et al., 1983). Between 90 and 135 minutes post-dose, subjects rested quietly in the scanner while six whole-brain rCBF maps were acquired using a pulsed-continuous ASL sequence (pCASL; Dai et al., 2008). In this method, blood from the neck and base of the brain is labeled using a train of Hanning-shaped radio frequency (RF) pulses of 500 μs duration, and a time gap of 1000 μs between each Hanning pulse. The total duration of the pulse train is 1.5 s (s). A sequence of gradient pulses of similar duration and repetition rate was employed to obtain flow-driven adiabatic inversion. The highest gradient amplitude under the Hanning pulses and the average gradient intensity over the RF train duration, were 9 mT/m and 1 mT/m, respectively. These values were originally chosen to ensure that the adiabatic condition for inversion and the exclusion of the first aliased labeling plane away from the excitation bandwidth of the Hanning pulse, were both met (Dai et al., 2008). In the control phase, the sign of alternate Hanning pulses was reversed, and the amplitudes of the gradient pulses were adjusted so that the net RF and gradient amplitudes over the 1.5 s irradiation were both zero. Thus, the magnetization transfer effect is compensated while achieving no inversion of arterial spins.

Image acquisition was performed using a 3D interleaved spiral fast spin echo (FSE) readout (Dai et al., 2008) with parameters: TR = 4 s, TE = 32 ms, ETL = 64, 8 interleaves, spatial resolution = 1 × 1 × 3 mm. Three ‘control-labeled’ pairs were collected to produce the ‘perfusion weighted’ difference image.

To quantify rCBF using this difference image, the sensitivity of the acquisition was calibrated to water at each voxel (Alsop and Detre, 1996; Buxton et al., 1998; Williams et al., 1992). This is complicated by the spatial non-uniform sensitivity of the 8-channel coil employed for this work. The underlying tissue signal is used as an indicator of water sensitivity, and a water density in each voxel, or partition coefficient, is assumed. In the original methodology (Dai et al., 2008), it was observed that the signal intensity in an inversion-prepared fluid-suppressed image was relatively constant for different tissues. This is likely because more complete recovery occurs for shorter T1 tissues, which tend to have lower water density. Using a neighborhood maximum algorithm to avoid regions with partial volume of suppressed fluid, a low resolution sensitivity map was created. This map was calibrated for water sensitivity by assuming the tissue was white matter with a water concentration of 0.735 g/ml (Herscovitch and Raichle, 1985) and a T1 of 900 ms, and using the equations for inversion recovery signal attenuation. By assuming that gray matter has a water concentration of 0.88 g/ml and a T1 of 1150 there was only a 5% calibration difference. This calibration produced a sensitivity map, C, equal to the fully relaxed MRI signal intensity produced by 1 g of water per milliliter of brain. With this co-registered sensitivity map C, we calculated cerebral blood flow (CBF) using the equation:

$$\text{CBF}=\frac{\rho_{b}({\text{S}}_{c}-{\text{S}}_{l})}{2\alpha \text{C}\omega_{a}\text{T}1_{a}\exp\left(-\frac{\text{δ}}{\text{T}1_{a}}\right)\left[1-\exp\left(-\frac{tl}{\text{T}1_{a}}\right)\right]}$$

where *ρ*_*b*_ is 1.05 g/ml (the density of brain tissue; Herscovitch and Raichle, 1985), α is the labeling efficiency (assumed to be 95% for labeling times 75% for background suppression; (Garcia et al., 2005), δ is 1.5 s (the post labeling delay; Alsop and Detre, 1996) *tl* is 500 ms (the labeling duration), T1_a_ is 1.4 ms (the T1 of arterial blood which was slightly lower than the value of Lu et al. (2004)), *ω*_*a*_ is 0.85 g/ml (the density of water in blood; Herscovitch and Raichle, 1985), S_*l*_ and S_*c*_ are the signal intensities in the labeled and control images, respectively. As is common in the ASL literature, this equation assumes that the labeled blood remains in the arterioles and capillaries and does not reach the tissue. The CBF quantification process does not alter the qualitative appearance of the images obtained by subtracting the label from the control image. The whole ASL pulse sequence, including the acquisition of calibration images, was performed in 6:08 min. After the acquisition of the pCASL scans, subjects performed a rewarded working memory task, which has been reported separately (Marquand et al., 2011). For each subject, a high-resolution T2-weighted FSE structural image was also acquired to assist registration of the pCASL scans to a common reference space with parameters: TR = 4.4 s, TE = 65 ms, FA = 90°, 36 × 4 mm thick oblique axial slices, in-plane resolution = 0.46 × 0.46 mm.

Images were preprocessed using tools from the Statistical Parametric Mapping 5 (SPM5; www.fil.ion.ucl.ac.uk) and Functional Software Library (FSL; www.fmrib.ox.ac.uk/fsl/) software packages. A three step procedure was employed to ensure maximally accurate registration of the pCASL image to a common reference image. First, extra-cerebral signal from the T2 structural scan was removed using the brain extraction tool included in FSL (BET; Smith, 2002) and the skull-stripped T2 image and its corresponding binary mask were co-registered to each pCASL image using SPM5. Second, the brain mask derived from the T2 image was applied to each pCASL image and the resulting skull stripped images were then co-registered back to the original T2 image (again with SPM5). Finally, the high resolution T2 image was used to compute SPM5 normalization parameters necessary to warp the image to the T2 MNI template provided with SPM5 and the resulting parameters were applied to the co-registered pCASL images in addition to the T2 image. Following normalization, each whole-brain pCASL image was spatially smoothed with an 8 mm isotropic Gaussian kernel and an average image was estimated for each subject and drug condition based on all scans. Since basal rCBF values are potentially different between participants, each image was then mean-centered within participants. In other words, a mean image was computed for each participant based on all images for that participant included in the classification problem and the mean was subtracted voxel-wise from each of the smoothed and averaged pCASL images. These mean-centered images were then reshaped into vectors and used as input to the classifiers.

### Sparse multinomial logistic regression classifiers

Sparse multinomial logistic regression (SMLR; Krishnapuram et al., 2005; Ryali et al., 2010) is the primary data analysis approach employed in this study. Like other multivariate PR techniques, SMLR holds two advantages over conventional mass-univariate techniques: (i) it is more sensitive for the detection of spatially distributed effects and (ii) it can make predictions at the level of individual subjects based on the pattern within the data. Another important feature of SMLR is that it is inherently formulated on a multi-class basis and can therefore discriminate between more than two classes simultaneously. Thus, it is more appropriate for the three-way classification problem posed in this study than a binary classification approach. In contrast, many alternative classification algorithms such as the support vector machine classifier (SVM; Scholkopf and Smola, 2002) are fundamentally limited to binary classification and only support multi-class classification via ad-hoc methods (e.g. decomposing the classification problem into binary sub-problems). In this study, we first applied a three-class classifier to discriminate the effect of MPH, ATX and PLC on rCBF. Then, to further investigate the differential effects of MPH and ATX, we trained a second binary classifier to directly discriminate between MPH and ATX.

A primary goal of this application is to find discriminating patterns of brain regions that permit accurate discrimination of each of the classes. To characterize these patterns as accurately as possible, it is important to restrict them to a parsimonious set of brain regions, which helps to prevent inferring that a brain region is necessary to discriminate classes when in fact it is not. In other words, we seek a sparse representation for the discriminating pattern. In a neuroimaging context, there are several approaches to achieve this, but two of the most common are feature selection approaches such as recursive feature elimination (RFE; Guyon et al., 2002; Hanson and Halchenko, 2008) and models employing regularization penalties that enforce sparsity (e.g. Carroll et al., 2009; Ryali et al., 2010; Yamashita et al., 2008). We adopt the latter approach in this paper, and following Ryali et al. (2010), we employ the elastic net penalty that combines an L1 penalty that enforces sparsity over the voxel coefficients with an L2 penalty that permits correlated voxels to be included. Inclusion of correlated voxels is important for neuroimaging data because (i) neuroimaging data are characterized by a high degree of spatial correlation and (ii) the spatial geometry of discriminating clusters is informative about the involvement of the underlying brain regions. In contrast, models that only employ L1 regularization penalties result in extremely sparse voxel sets that are not informative about the spatial geometry of discriminating clusters (Yamashita et al., 2008) and can yield classifiers that generalize poorly (Marquand et al., 2010). In contrast, for binary classification, SMLR with an elastic net penalty is known to more accurately identify discriminating voxels relative to SVM with RFE while producing equivalent classification accuracy (Ryali et al., 2010).

We provide a brief description of SMLR here and refer the reader elsewhere for a detailed treatment (Krishnapuram et al., 2005; Ryali et al., 2010). We denote the training dataset by *D* = {**X**,**Y**}, where **X** is an *n* × *d* matrix with the *d*-dimensional data vectors (**x**_*i*_) stacked in rows and **Y** is an *n* × *m* matrix that describes the labels for the *m* classes. We adopt a ‘one-of- *m*’ coding scheme where *y*_*ij*_ = 1 if sample *i* belongs to class *j* and zero otherwise. The starting point for classification is a multinomial likelihood function which models the probability of assigning a data sample to each class using a softmax transformation. Thus, the probability of data sample **x**_*i*_ belonging to class *j* is given by:

(1) $$p(y_{\mathrm{ij}}=1|x_{i})=\pi_{\mathrm{ij}}=\frac{\exp(w_{j}^{\text{T}}x_{i})}{\sum_{k=1}^{m}\exp(w_{k}^{\text{T}}x_{i})\text{.}}$$

Here, **w**_*j*_ denotes a d-dimensional vector of voxel weights predictive of class *j* and to keep the notation concise, we concatenate the weight vectors for all classes into a *dm*-dimensional weight vector **w**. This formulation leads to a convenient form for the log-likelihood of the entire dataset, i.e.:

(2) $$\begin{array}{c} L(w)=\log\prod_{i=1}^{n}\prod_{j=1}^{m}\pi_{\mathrm{ij}}^{\mathrm{yij}}\\ =\sum_{i=1}^{n}\sum_{j=1}^{m}y_{\mathrm{ij}}\log\pi_{\mathrm{ij}}\\ =\sum_{i=1}^{n}\sum_{j=1}^{m}y_{\mathrm{ij}}w_{j}^{\text{T}}x_{i}-\sum_{i=1}^{n}\log\sum_{j=1}^{m}\exp(w_{j}^{\text{T}}x_{i})\text{.} \end{array}$$

We then apply the elastic net regularization penalty to Eq. (2) which enforces sparsity over voxels and helps prevent overfitting by constraining the magnitude of the weights. Thus, the objective function we need to maximize is:

(3) $$J(w)=L(w)-\lambda_{1}||w|{|}_{1}-\lambda_{2}||w|{|}_{2}$$

where *λ*_1_ and *λ*_2_ are parameters that respectively control the degree of L1 and L2 regularization. In this paper, we employ an efficient component-wise update algorithm to optimize Eq. (3), which has been described in detail elsewhere (Krishnapuram et al., 2005). We employed nested cross-validation with a grid search to find optimal values for the regularization parameters as described in the next section. Once the optimal weight vectors have computed, we make predictions by applying Eq. (1) to the scans derived from the test subject. This yields a probabilistic prediction for each class, which can be converted to categorical predictions by simply choosing the class having the highest probability.

### Cross-validation

We employed nested leave-one-subject-out cross-validation (LOO-CV) to simultaneously evaluate the generalization ability of the classifier and find optimal values for the regularization parameters *λ*_1_ and *λ*_2_. In an outer LOO-CV loop, we excluded all scans from a single subject to form the test set and in an inner LOO-CV loop, we repeatedly partitioned remaining subjects into a validation set (1 subject) and training set (13 subjects), excluding each subject once. This provides a relatively unbiased estimate of generalization ability derived only from the training set which can be used to find the optimal parameter settings for the subject held out in the test set. To achieve this, we varied *λ*_1_ and *λ*_2_ logarithmically across a wide range of values (from 10^− 5^ to 10^5^ in steps of 10). We then selected the values for *λ*_1_ and *λ*_2_ that yielded maximum LOO-CV accuracy on the validation set for prediction on the test set. The grid search yielded well-peaked optimal parameter settings which were also stable across outer LOO-CV folds (*λ*_1_: mean = 0.03, SEM = 0.01; *λ*_2_: mean = 1.82, SEM = 0.87). To estimate generalization ability for each classifier, we measured the predictive accuracy for each class by counting the number of class labels correctly predicted on the test set and averaging over all outer loop LOO-CV folds. Finally, we averaged these class accuracies over all classes to derive an overall measure of classification accuracy.

### Multi-class discrimination maps

One of the benefits of employing a multi-class classification approach is that it provides a spatial representation of the discriminating pattern for each class. This approach is the multi-class generalization of discrimination mapping (Mourao-Miranda et al., 2005), which has to date most commonly been performed in a binary classification context although an L1-regularized SMLR approach has been used previously for multi-class discrimination mapping (Yamashita et al., 2008). Exactly as in the binary context, SMLR weight vector coefficients encode the contribution of each voxel to the decision function for each class relative to all the other classes. Thus, a high positive score in the weight vector for a given class denotes a strong positive contribution to a prediction in favor of that class, while a high negative score for the same class denotes a strong negative contribution. To explain this more clearly, note that to determine the predicted label it is necessary to consider the relative intensity of voxel values in addition to the sign and magnitude of the weights. Thus, voxels with negative weight vector coefficients can contribute positively to the decision for the weight vector's class if the voxel intensities of brain images corresponding to that class are lower than the other classes.

For this application, we are primarily interested in the differential activity patterns for MPH and ATX with respect to the PLC class, which can be considered a reference class. To facilitate interpretation of the weight vectors, it is therefore convenient to visualize the relative difference between each of the drug classes and PLC instead of each weight vector independently. This can be achieved by first noting that the multinomial likelihood given in Eq. (1) is redundant because the class probabilities must sum to one (i.e. *Σ*_*j* = 1_^*m*^*π*_*ij*_ = 1), thus without loss of generality an equivalent reparameterisation of the classification problem can be derived by fixing one of the weight vectors to zero (in this case, the PLC class, which we denote by **w**_*m*_). Under this reparameterisation, the weight vectors for the other two classes are given by:

(4) $${w^{\prime }}_{j}=w_{j}-w_{m}\text{.}$$

The discrimination maps presented in this paper for the multi-class classifier are spatial representations of the weight vectors specified by Eq. (4).

To aid interpretation of the SMLR discrimination maps, we also compute conventional statistical parametric maps (SPMs) that quantify the magnitude and indicate the direction of focal effects in each brain region. This is important for three reasons: (i) the direction of rCBF changes cannot be directly determined from the weight vector alone, (ii) as noted above, it is necessary to consider the relative intensity of each class to correctly interpret the weight vector and (iii) multivariate discrimination maps describe a pattern of changes potentially distributed across many brain regions and do not describe regionally specific effects. Thus, in addition to the SMLR weight vectors, we computed a simple unpaired t-statistic for each voxel using the same data that was used to train the classifier. Note that we employed an unpaired *t*-test to most accurately approximate the behavior of the classifier, although similar results were obtained using a paired *t*-test. Further, we present unthresholded maps since it is necessary to quantify the magnitude of regional changes in all brain regions, not only in those surviving an arbitrary univariate threshold.

## Results

### Classification accuracy for SMLR classifiers

**Fig. 1:**
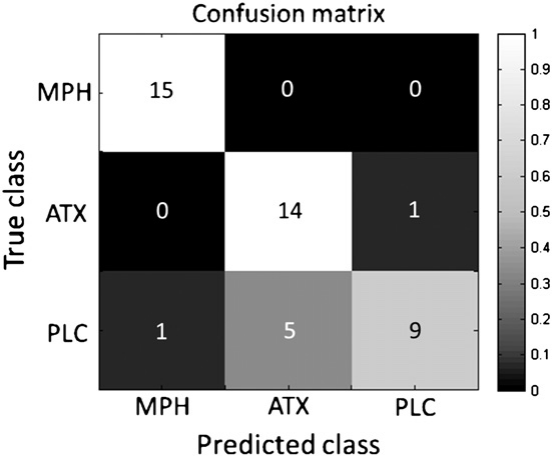
Confusion matrix for multiclass classifier contrasting MPH, ATX and PLC. The color scale indicates proportion of correct predictions and the numerals superimposed describe the number of correct predictions for each cell (out of a maximum of 15 per class).

The multiclass SMLR classifier trained to discriminate between all drug conditions correctly classified 100.00% of MPH scans, 93.33% of ATX scans and 60.00% of PLC scans, leading to an overall accuracy of 84.44%, easily exceeding the 33.33% accuracy that would be predicted by chance (*χ*^2^ = 39.40, p = 2.78 × 10^− 9^). A confusion matrix derived from this classifier (Fig. 1) indicates that: (i) the only misclassification from the ATX scans was an erroneous prediction for PLC and (ii) most misclassifications of the PLC scans were erroneous predictions for ATX although one PLC scan was erroneously predicted as MPH.

The separate, binary SMLR classifier trained to discriminate between MPH and ATX correctly classified 93.33% of MPH and 93.33% of ATX scans, yielding an overall accuracy of 93.33% which again exceeded the 50% accuracy that would be predicted by chance (p = 2.89 × 10^− 8^, binomial test).

**Fig. 2:**
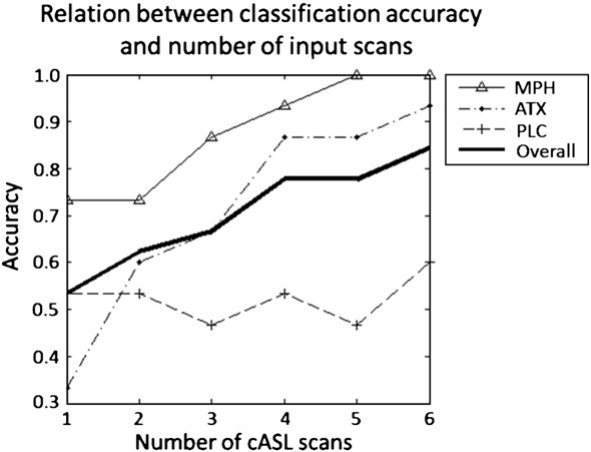
Classification accuracy plotted as a function of the number of pCASL scans used to train the classifier.

When the number of pCASL scans used to train the classifier was varied, the overall classification accuracy increased monotonically with increasing number of scans (Fig. 2). Note that this effect was largely restricted to the drug conditions; discrimination accuracy for PLC remained relatively constant (i.e. 53.33% for the first scan and 60.00% for all six scans). Across all scan numbers the classification accuracy for ATX was consistently lower than MPH with a large difference in discrimination accuracy with only one scan included (5/15 versus 11/15 correct predictions respectively). This emphasizes the importance of acquiring multiple pCASL scans because when all six scans were included, very similar accuracies were obtained for ATX and MPH (14/15 versus 15/15 correct predictions respectively).

### Discrimination maps for multiclass SMLR classifier

**Fig. 3:**
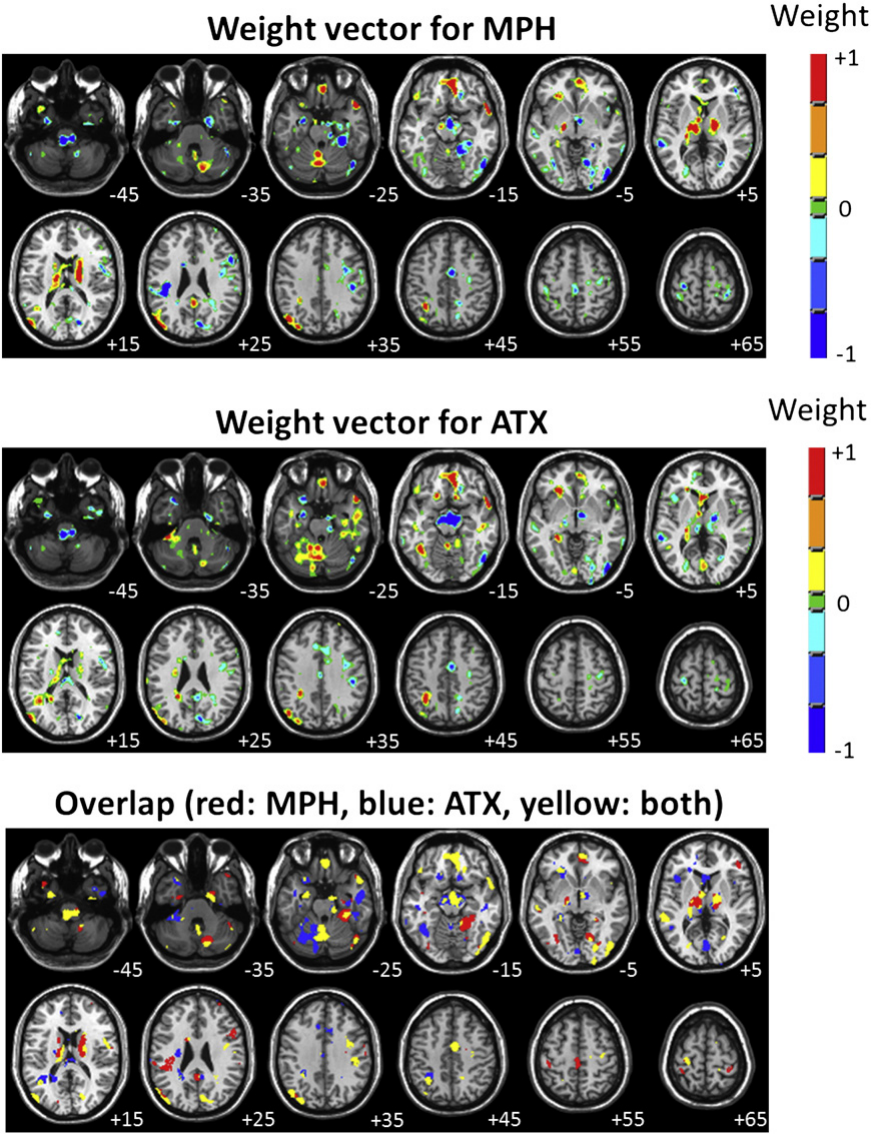
SMLR weight vector discrimination maps for the multi-class classifier discriminating between all drug conditions. Top panel: weight vector for MPH, middle panel: weight vector for ATX, bottom panel: overlapping voxels. For the top two panels, positive coefficients (red color scale) indicate a positive contribution to the prediction for each class and negative coefficients (blue color scale) indicate a negative contribution. For the bottom panel, red indicates voxels with non-zero coefficients in the MPH weight vector, blue indicates voxels with non-zero coefficients in the ATX weight vector and yellow indicates voxels with non-zero coefficients in both weight vectors. Note that the weight vector for PLC is fixed to zero and that the scale for weight vector coefficients is arbitrary. The right hand side of each image corresponds to the participants' right side and numerals in white text indicate Z-coordinates in Talairach space.

Spatial representations of the SMLR weight vectors derived from the multi-class classifier are presented in Fig. 3 (top two panels). To assist interpretation, weight vectors are presented for MPH and ATX only, using the PLC class as a reference (see Methods). As noted, SMLR weight vector coefficients encode the contribution of each voxel to the decision function for each class, thus they may be interpreted as spatially distributed patterns of brain regions with predictive value for each drug with respect to PLC. For example, high positive weights for MPH in a given brain region have predictive value for MPH with respect to PLC, while high negative weights indicate predictive value for PLC with respect to MPH. To assist visualization, a map showing the overlap between voxels having non-zero coefficients in the MPH and ATX weight vectors is also presented in Fig. 3 (bottom panel).

The predictive patterns for MPH and ATX were both moderately sparse. The predictive pattern for MPH contained clusters of positive coefficients encompassing cortical and subcortical brain regions including bilateral caudate body, thalamus, midbrain/substantia nigra (SN), ventromedial prefrontal cortex (vmPFC), temporal poles, left superior parietal lobe and right cerebellum. Negative coefficients were mostly cortical with clusters in right lateral frontal, mid-cingulate and sensorimotor cortex, amygdala, parahippocampal gyrus and in multiple regions of occipital, temporal cortex. In addition, relatively large clusters of negative coefficients were found in the pedunculo–medulla boundary and midbrain/hypothalamus.

The predictive pattern for ATX also encompassed widespread brain regions and its most notable feature was a large cluster of negative coefficients centered in the midbrain, in the region of the SN and hypothalamus, extending dorsally to the right thalamus. Clusters of negative coefficients were also found in sensorimotor cortex, mid-cingulate, amygdala, parahippocampal gyrus, pedunculo–medulla boundary and small regions of occipital and temporal cortex. Clusters of positive coefficients were found in vmPFC, right temporal pole, left superior parietal lobe, left cerebellum and regions of right temporal cortex.

### Discrimination map for binary SMLR classifier contrasting MPH and ATX

**Fig. 4:**
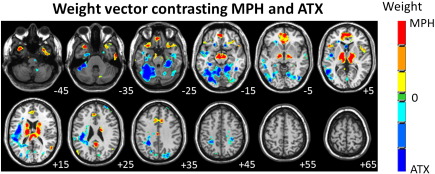
SMLR weight vector discrimination map for the binary classifier contrasting MPH and ATX. Positive coefficients (red color scale) indicate a positive contribution to the prediction of MPH and negative coefficients (blue color scale) indicate a positive contribution to prediction of ATX. The scale for the weight vector coefficients is arbitrary and the right hand side of each image corresponds to the participants' right side and numerals in white text indicate Z-coordinates in Talairach space.

A spatial representation of the SMLR weight vector derived from the binary classifier contrasting MPH and ATX is presented in Fig. 4. In this case, positive coefficients denote regions having predictive value for MPH and negative coefficients denote regions having predictive value for ATX. This pattern showed a good overall correspondence with those derived from the multiclass classifier in that: (i) clusters of coefficients predictive for MPH included bilateral caudate body, midbrain/SN, thalamus, vmPFC, cingulate cortex, insula and temporal poles as well as small regions in the inferior frontal gyrus, middle and inferior temporal gyri and cerebellum (ii) clusters of coefficients predictive for ATX were mainly localized to cerebellum, parahippocampal gyrus, posterior insula, middle and inferior frontal gyri, sensorimotor cortex, middle temporal gyrus and small regions of occipital cortex.

### Univariate statistical parametric maps

**Fig. 5:**
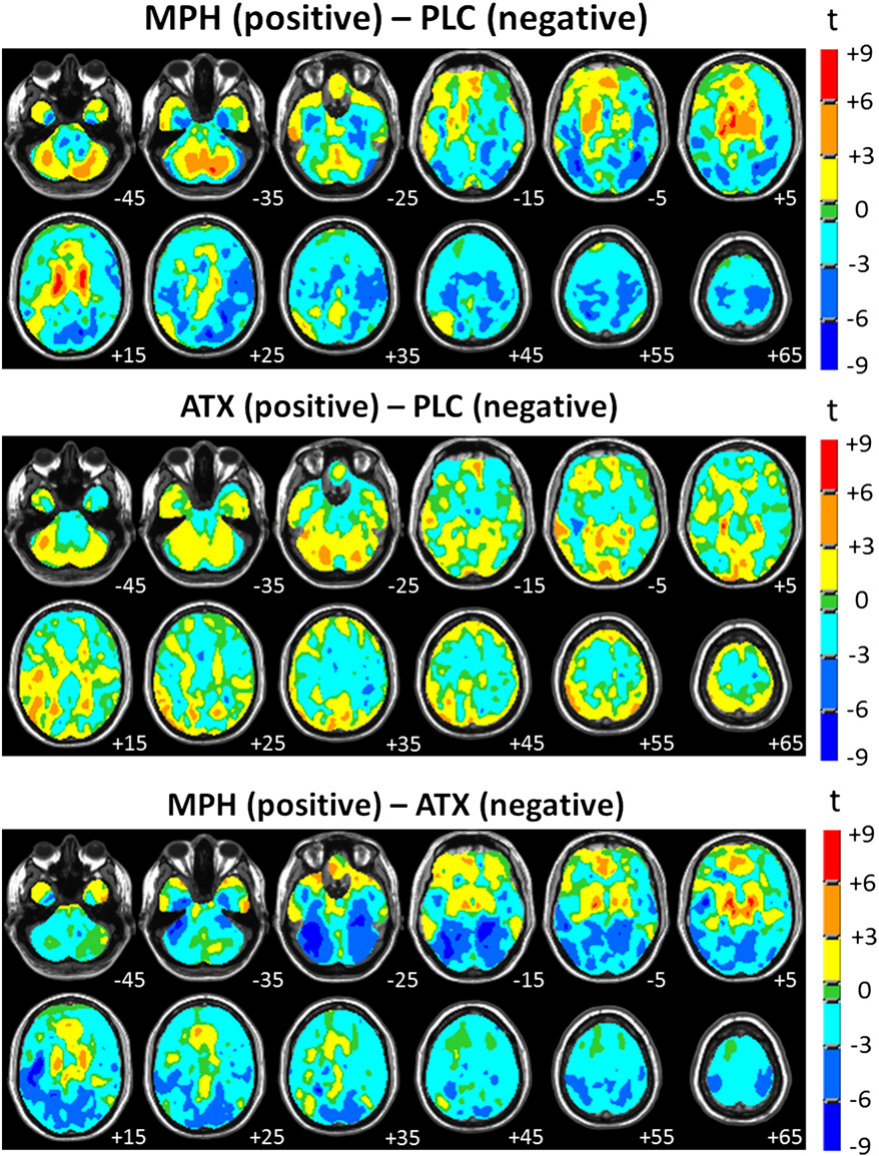
Univariate t-statistic statistical parametric maps for each binary contrast. Note that maps are not thresholded to facilitate interpretation of SMLR weight vector maps. The right hand side of each image corresponds to the participants' right side and numerals in white text indicate Z-coordinates in Talairach space.

Univariate statistical parametric maps (SPMs) were computed for each binary contrast and are presented in Fig. 5. All SPMs show a good correspondence with the discrimination maps described above in that: (i) most regions with high magnitude weight vector coefficients also have a high magnitude t-statistics (positive or negative) and (ii) the direction of weight vector coefficients and t-statistic agrees in nearly all regions.

## Discussion

In this study, we employed pCASL and multi-class pattern recognition to accurately discriminate the effects of single acute doses of MPH, ATX and PLC on rCBF in healthy human volunteers. We demonstrated that drug discrimination accuracy increased monotonically with increasing number of pCASL scans, suggesting that pharmacological studies that utilize a single pCASL scan may be suboptimal. We also presented discriminative activity patterns for each drug relative to PLC and a pattern that directly discriminated between MPH and ATX, which collectively identified their differential effects across widespread brain regions. This study extends existing MPH studies by providing evidence that pCASL is sufficiently sensitive to detect similar drug effects to those observed in existing H_2_[O^15^] and [^18^F]DG PET studies and to our knowledge, represents the first attempt to characterize the effects of ATX on human rCBF at rest. More generally, our results provide insight into the regional distribution of the brain networks potentially modulated by these compounds.

Several features of the predictive pattern we derived for MPH correspond with effects of MPH identified by previous PET studies. It is important to note however, that the scope of comparison with existing studies is constrained by the different analysis methods employed. That is, the use of univariate approaches in the existing literature is limited to reporting local changes exceeding a predefined statistical threshold, whereas the multivariate approach used in this study finds a pattern of regions that optimally discriminates between drug classes. The most consistently reported regions modulated by MPH in existing H_2_[O^15^] and [^18^F]DG PET studies were the cerebellum and temporal poles (Mehta et al., 2000; Udo de Haes et al., 2007; Volkow et al., 1997), although as noted, the direction of rCBF changes in the temporal poles varies between studies. Despite the limitations of comparison we have highlighted it is noteworthy that these were prominent features of the predictive pattern for MPH in our analysis. Another prominent feature was increased rCBF in the caudate body, congruent with multiple lines of evidence showing that MPH increases striatal DA concentrations (e.g. Bymaster et al., 2002; Volkow et al., 1994), although the striatum was not observed following methylphenidate challenge in human H_2_[O^15^] PET studies (Mehta et al., 2000; Udo de Haes et al., 2007). Indeed, there is emerging evidence that H_2_[O^15^] PET may show sensitivity only for larger rCBF changes in the striatum (Borghammer et al., 2009) and that subcortical effects show greater variability in H_2_[O^15^] and [^18^F]DG PET studies relative to cortical effects (Ma et al., 2009).

Many, but not all features of the pattern of rCBF changes we observed following MPH can be related to catecholamine transporter distribution: in humans and other primates, the caudate and thalamus have high DAT density (Ciliax et al., 1999; Garcia-Cabezas et al., 2007; Sanchez-Gonzalez et al., 2005), the cerebellar vermis has moderate DAT density (Melchitzky and Lewis, 2000) and virtually the entire cerebellum receives a rich noradrenergic innervation (Powers et al., 1989). Relative to other brain regions, cocaine shows high levels of binding in human temporal pole, indicating relatively high monamine transporter density (Telang et al., 1999). In addition, it is known that NAT has a more prominent role in DA uptake in the prefrontal cortex relative to DAT (Kaenmaki et al., 2010; Moron et al., 2002) and thus the medial prefrontal cortex rCBF changes for MPH, as well as ATX, align with the functional role of NAT in DA clearance in this region. It is difficult to definitively assign clusters in the midbrain and brainstem to specific nuclei owing to the small size of such nuclei relative to the resolution of the pCASL images acquired, but the pattern predictive of MPH contains clusters of positive coefficients that are relatively well localized to the SN bilaterally. Clusters of negative coefficients reflecting decreased perfusion in the pedunculo–medulla boundary, hypothalamus and amygdala are also consistent with a population of noradrenergic cells in the nucleus of the solitary tract (NST) that projects to the hypothalamus, amygdala and other limbic structures via the ventral noradrenergic bundle (Cunningham and Sawchenko, 1988; Moore and Bloom, 1979).

The slightly lower predictive accuracy for ATX relative to MPH combined with the moderately high rate of erroneous predictions of PLC scans for ATX suggests that the neuronal effects of ATX were slightly weaker than those of MPH at the doses administered, although the class accuracies for both ATX and PLC remained well above chance level. Similar to MPH, the predictive pattern we derived for ATX included several regions having high NAT density including the thalamus, cerebellum and sensorimotor cortex (Ghose et al., 2005; Hannestad et al., 2010; Powers et al., 1989; Schou et al., 2005; Tejani-Butt, 1992). The pattern for ATX also included clusters of negative coefficients in the midbrain/hypothalamus, amygdala, and pedunculo–medulla boundary overlapping those observed following MPH, suggesting that ATX also influenced noradrenergic projections emerging from the NST.

The pattern discriminating between MPH and ATX and the SPM derived from the same contrast showed that the clearest differential effects of MPH and ATX were in the caudate body, thalamus, midbrain/SN and cerebellum although the overall pattern was distributed across widespread brain regions congruent with the effects noted above. The SPM indicates that discriminative clusters in the caudate body reflect increased rCBF for MPH relative to ATX and are thus consistent with rodent microdialysis results that show that MPH but not ATX increases extracellular DA concentration in the striatum (Bymaster et al., 2002). Similarly, the SPM indicates that discriminative clusters in the thalamus and midbrain/SN reflect opposing effects of MPH and ATX, where in both regions, MPH increases rCBF while ATX decreases it. Further, the rCBF decreases in the midbrain/SN produced by ATX are amongst the strongest focal effects of the drug in any brain region. Overall, these effects are consistent with several lines of evidence from studies with experimental animals indicating that noradrenergic mechanisms influence nigrostriatal DA release (e.g. Grenhoff et al., 1993; Lategan et al., 1990, 1992; Marien et al., 2004), but a paucity of evidence for direct projections from noradrenergic cell populations to the SN suggests that such effects are likely to be mediated indirectly (Marien et al., 2004; Swanson and Hartman, 1975). Our data suggest the thalamus as a candidate region for mediating this effect. In the cerebellum, the spatial distribution of rCBF changes was clearly different for the two drugs, where ATX decreased rCBF across widespread regions of cerebellar cortex relative to MPH. This suggests that striato-cerebellar circuitry may be differentially affected by MPH and ATX and makes the prediction that cognitive and behavioral functions subserved by the striato-cerebellar network may be particularly sensitive to the differential effects of MPH and ATX.

Despite the correspondence of many components of the predictive patterns for MPH and ATX with the regional distribution of catecholamine transporters, it is important to emphasize that the overall distribution of each pattern would have been difficult to predict solely on the basis of regional transporter density. For example, the predictive pattern for ATX did not include the LC despite the LC having the highest NAT density in the brain (Schou et al., 2005; Tejani-Butt, 1992). Thus, the functional consequences of increased catecholaminergic neurotransmission are not only expressed in regions of high transporter density but also in connected brain areas. This suggests that the effects of increased noradrenergic transmission in the LC are probably expressed distally (e.g. in the thalamus), while those of other noradrenergic cell populations (e.g. that of the NST) are expressed more proximally.

An advantage of ASL is that it allows direct comparison of the underlying regional perfusion across multiple visits, which is not possible using BOLD fMRI because the BOLD signal is not quantitative. Further, and as noted above, the BOLD signal depends on rCMRO_2_ and rCBV in addition to rCBF, which complicates the interpretation of studies that have employed BOLD fMRI to investigate the effects of MPH and ATX on brain activation during cognitive tasks in healthy volunteers (e.g. Chamberlain et al., 2009; Dodds et al., 2008; Graf et al., 2011; Marquand et al., 2011) and patients with ADHD (e.g. Rubia et al., 2011; Schweitzer et al., 2004; Vaidya et al., 1998). Our results show that ASL can accurately describe the pattern of baseline rCBF changes for MPH as well as ATX, which is useful to identify the contribution of rCBF changes to the BOLD response in different brain regions and will therefore provide a more precise neurophysiological understanding of how these drugs modulate brain function. In addition, the sensitivity of BOLD to signal drop-out and/or cardiac-pulse-induced artifacts in many of the brain regions observed to be differentially affected by MPH and ATX (e.g. temporal pole and brainstem) implies that ASL may be better suited than BOLD fMRI to detect the effects of these drugs in these regions. Finally, it is important to consider that DA and NA are both vasoconstrictive agents (Krimer et al., 1998; Mulligan and MacVicar, 2004; Raichle et al., 1975), meaning that we cannot quantify the degree to which vascular effects contributed to the pattern of changes we report for MPH and ATX.

A limitation of this study is that only a single dose of each drug was administered so we cannot exclude dose effects explaining some aspects of the differential rCBF pattern for MPH and ATX, although this would appear to be unlikely for three reasons: first, administered doses were matched according to doses commonly used in clinical practice. Second, motor evoked potentials and task-related activations and deactivations are altered to a similar extent for both drugs using identical doses to those administered here (Gilbert et al., 2006; Marquand et al., 2011). Third and most importantly, opposing effects of MPH and ATX in overlapping brain regions (e.g. the midbrain/SN) are difficult to explain by a simple dose effect.

In conclusion, we have demonstrated common and differential effects of MPH and ATX on rCBF in healthy volunteers at rest using multi-class pattern recognition. This methodology differs from classical univariate analyses in that the latter would only show areas where the amplitude of rCBF change was large between drug conditions, rather than spatially distributed activity patterns. Our results show that the effects of MPH and ATX overlapped in multiple, distributed brain regions and had clearly differential effects in striato-cerebellar circuits, the thalamus and in the midbrain/SN. Further, we showed the sensitivity of pattern recognition methods in detecting an acute dose of MPH and ATX which illustrates the feasibility of predicting the effects of such medications (e.g. treatment response) at the level of individual subjects.
